# Health Literacy Perceptions and Knowledge in Pediatric Continuity Practices

**DOI:** 10.3928/24748307-20220208-01

**Published:** 2022-01

**Authors:** Elaine Griffeth, Iman Sharif, Alexandria Caldwell, M. Townsend Cooper, Hollyce Tyrrell, Marny Dunlap

## Abstract

**Background:**

Low health literacy affects one-third of adults in the United States and can have a negative effect on health behavior and outcomes.

**Objective:**

The goal of this study was to examine attitudes and knowledge of health literacy among pediatric residents and faculty in pediatric resident continuity clinics across the country.

**Methods:**

An online mixed-methods survey was distributed to pediatric faculty and residents through the Academic Pediatric Association's Continuity Research Network. The 20-question survey included questions about the participants' health literacy knowledge and health literacy practices in continuity clinics, such as use of universal health literacy precautions. Categorical answer choices were dichotomized into positive and negative groupings and resident and faculty responses were compared using the Chi-squared test (significance *p* < .05). Qualitative data were analyzed using emergent coding and grounded theory to determine themes.

**Key Results:**

Responses were received from 402 individuals at 24 pediatric residency programs. Most participants agreed or strongly agreed that they could correctly identify participants with low health literacy (76% residents vs. 53% faculty). Only 19% of residents and 26% of faculty were familiar with universal health literacy precautions. Many residents and faculty had received no training in health literacy (37% residents vs. 38% faculty). Barriers and challenges around health literacy included time, language, limited training or resources, low literacy, disease mismanagement, and fixed misconceptions.

**Conclusion:**

Despite ample evidence in the literature to the contrary, most respondents believed they could correctly identify individuals with low health literacy. Additionally, most participants had not heard of universal health literacy precautions and were unaware of their usage in their practice setting. This is not consistent with current expert recommendations. These findings are troubling as they are from academic residency programs, indicating an educational deficit. These findings point toward a next step in health literacy education for pediatric residents. [***HLRP: Health Literacy Research and Practice*. 2022;6(1):e51–e60.**]

**Plain Language Summary:**

Pediatric residents and faculty in continuity clinics were surveyed about their opinions, health literacy knowledge, ability, and practices in continuity clinics. Despite evidence to the contrary, most respondents believed they could correctly identify individuals with low health literacy and had not heard of universal health literacy precautions. These findings highlight the need for more health literacy education for pediatric residents.

Health literacy is defined by the Institute of Medicine as: “The degree to which individuals have the capacity to obtain, process, and understand basic health information and services needed to make appropriate health decisions” (Nielsen-Bohlman et al., 2004). Low health literacy affects approximately one-third of adults in the United States (Kutner et al., 2006). Many studies have examined the health literacy of parents in the U.S. and show limited health literacy and ability to correctly perform various health-related tasks across a variety of populations (Leyva et al., 2005; Lo et al., 2006; Tran et al., 2008; Yin et al., 2009; Yin et al., 2016). Research also has shown that low health literacy skills in adult patients and among pediatric caregivers correlate with worse health behaviors and poorer health outcomes (Berkman et al., 2008; Keller et al., 2008; Kumar et al., 2010; Mancuso & Rincon, 2006; Miller et al., 2010). Limited parental health literacy has been linked with increased perceived barriers to access care and negative parental perceptions of their own ability to care for chronic illness (Wood et al., 2010; Yin et al., 2012). The Joint Commission (n.d.) has also maintained that unaddressed health literacy concerns pose a safety risk to patients and have embedded health literacy concepts into several of their requirements. These include providing information in a manner that the patient can understand, providing written information in plain language, and patient participation in care discussions. They highlight the “Teach Back” method as an easy-to-implement strategy to address patient understanding and improved decision-making about their health care (Cordero, 2018; Glick et al., 2019).

Unfortunately, studies have also shown that not only do physicians have difficulty identifying caregivers or patients with limited health literacy, but pediatricians feel they have inadequate communication skills and time to meet the needs of these caregivers (Bass et al., 2002; Chesser et al., 2012; Cooper et al., 2018; Kelly & Haidet, 2007; Turner et al., 2009). Additionally, research shows that residents overestimate the clarity with which they communicate and instead often use medical jargon and rarely use clear communication strategies such as Teach-Back techniques (Howard et al., 2013). Medical trainees perceive that they do not receive adequate training to feel confident in communicating with patients and caregivers with low health literacy (Ali et al., 2014). The Health Literacy Universal Precaution Tool-kit is recommended by the Agency for Healthcare Research and Quality (AHRQ) to ensure that comprehension is a primary goal with each patient, regardless of physician assessment of literacy level (Paasche-Orlow et al., 2006). Efforts have been made to promote the Health Literacy Universal Precautions as standard practice, but it is well documented that the medical community is still falling short of this goal (Liang & Brach, 2017).

There is a potential gap in pediatric graduate medical education regarding health literacy, but this gap is not unique to pediatrics. Internal medicine and family medicine programs recognized a similar deficit and have implemented health literacy training curricula with positive results (Coleman et al., 2016; Green et al., 2014; Pagels et al., 2015). Several organizations, including the U.S. Department of Health and Human Services, have recognized the importance of health literacy in patient care and named physician training on low health literacy as a priority (Office of Disease Prevention and Health Promotion, 2020). Although health literacy is not currently explicitly stated as an Accreditation Council for Graduate Medical Education (ACGME) required competency, a health literacy curriculum covering health literacy knowledge and effective communication strategies would support several ACGME competencies, including the ability to counsel patients and families; participating in the education of patients and families; communicating effectively with patients, families, and the public, as appropriate, across a broad range of socioeconomic and cultural backgrounds; and demonstrating sensitivity and responsiveness to a diverse patient population, including but not limited to diversity in gender, age, culture, race, religion, disabilities, and sexual orientation (ACGME, 2020).

Although general guidelines and content recommendations exist and can aid in the development of this curriculum, it is important to first understand the extent and characteristics of health literacy training in U.S. pediatric residency programs (Coleman et al., 2013; Turner et al., 2009). The goal of this study was to examine attitudes and knowledge of health literacy among pediatric residents and faculty in pediatric resident continuity clinics across the U.S. Because of the academic context of the population, we hypothesized that the majority of faculty would have received some training on health literacy and would report that they use strategies and tools to improve communication with all patients. However, we hypothesized that residents would be less likely to report specific health literacy training or use of strategies to improve communication.

## Methods

Practices were recruited through the Academic Pediatric Association's Continuity Research Network (CORNET) from March 2019 until March 2020. At the time of the study, there were 125 pediatric training programs enrolled in CORNET, which includes more than 50% of all pediatric residency training programs nationally (Sharif & Tyrrell, 2019). Among the enrolled pediatric training programs are more than 160 individual continuity clinic practice sites and more than 5,900 categorical pediatric residents and medicine-pediatric residents who provide care to more than 1 million pediatric patients. A message describing the study was distributed to CORNET members via an electronic listserv. CORNET site champions responded with interest, then completed a brief enrollment survey where they provided the total number of continuity clinic preceptors and residents for their residency program. A CORNET site champion is the coordinator between their pediatric residency program (and associated continuity clinics) and CORNET. This individual is the main point of contact for CORNET, enrolls their program in CORNET studies, and ensures the program's membership profile in CORNET is kept up to date. When the site champion is not able to participate or lead a study's implementation at their program, they will appoint a colleague to do so. After enrollment, an online, anonymous research survey, developed with Research Electronic Data Capture, was sent to each CORNET site champion to distribute to the residents and faculty at their residency program. Consent was implied by survey completion. CORNET site champions received up to two email reminders. This study was approved by the University of Oklahoma Health Sciences Institutional Review Board.

The online, anonymous research survey was a 20-item electronic survey structured with branching logic such that additional follow-up questions were presented based on participants' responses. The survey gathered general information about the participants including their position (faculty, resident, other) and years in that role, continuity clinic practice setting (private practice, hospital-based practice, federally qualified health center [FQHC], community health center [not FQHC], military base, other), and the zip code of their practice. We asked health literacy knowledge and perceived ability questions adapted from evaluations developed by the Centers for Disease Control and Prevention and a continuing education online course on health literacy developed by Mackert et al. (2011) and the Centers for Disease Control and Prevention (2018). We also asked questions about health literacy practices in their continuity clinics such as the use of health literacy screening tools, universal precautions, and the American Academy of Pediatrics (2019) recommended techniques for communication with patients and caregivers with limited health literacy. Additionally, we asked questions about whether each respondent's institution provides health literacy education, their perception of the usefulness of education on health literacy, and if their institution has a faculty authority on health literacy. We also asked two qualitative questions: “What have been some unexpected challenges in addressing health literacy as part of your practice?” and “Please elaborate on an experience with a patient/caregiver where health literacy was a barrier and you felt that it impacted patient care.” Participants responded to these open-ended questions through anonymous free text responses.

## Analyses

Because this was a descriptive study, we powered this study to describe the 125 CORNET practices nationally, which include a total of approximately 7,500 faculty and residents. Using a 95% confidence level and setting a confidence interval of ±5 for the point estimate on training in health literacy, we planned to recruit a sample size of at least 365 participants. If we recruited at least 85 faculty, the sample size would also give us 80% power to detect at least a 15-point difference in estimates between faculty and residents, setting alpha at 0.05.

Response rate was calculated both on the program and individual level. For the individual level calculation, total program enrollment and faculty count at participating sites was used as the denominator. Respondents with insufficient data for analysis (missing basic demographics such as resident/faculty status) were noted and excluded. Basic descriptive statistics of the sample were calculated and reported. Geographic range was determined by mapping participant zip code.

Univariate analysis for all responses was performed. For several key outcomes, the variable responses were grouped to produce a binary outcome (affirmative vs. neutral or other). These outcomes were as follows: identification of care-giver low health literacy, familiarity with universal health literacy precautions, use of health literacy assessment tool, and receipt of training in health literacy. Dichotomized responses between the resident and faculty groups were compared using the Chi-squared test (significance level *p* < .05). All data analysis was done using the R statistical package. For the two qualitative questions, participant responses were coded by three researchers (A.C., M.D., and M.T.C.). Emergent coding employing grounded theory was used with holistic perspective (Patton, 2002). Each researcher (A.C., M.D., and M.T.C.) coded responses independently, then coded data were discussed among all three researchers (A.C., M.D., and M.T.C.) until consensus was reached. Coded data were then organized into themes and reviewed within each theme to ensure validity in the findings.

## Results

Of the 125 CORNET member residency programs, 24 (19%) agreed to participate and subsequently sent the survey to their residents and faculty. These programs contained 1,381 residents and 331 continuity clinic preceptors. There were 249 (18%) residents and 153 (46%) faculty that responded to the survey. Two respondents did not indicate resident or faculty status and were excluded from further analysis. **Table 1** contains specific details regarding respondents.

**Table 1:**
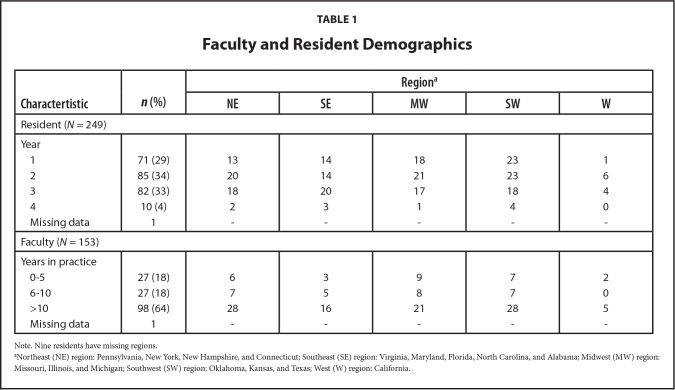
Faculty and Resident Demographics

**Charactertistic**	***n* (%)**	**Region^a^**				

		**NE**	**SE**	**MW**	**SW**	**W**

Resident (*N* = 249)						

Year						
1	71 (29)	13	14	18	23	1
2	85 (34)	20	14	21	23	6
3	82 (33)	18	20	17	18	4
4	10 (4)	2	3	1	4	0
Missing data	1	-	-	-	-	-

Faculty (*N* = 153)						

Years in practice						
0-5	27 (18)	6	3	9	7	2
6-10	27 (18)	7	5	8	7	0
>10	98 (64)	28	16	21	28	5
Missing data	1	-	-	-	-	-

Note. Nine residents have missing regions.

a Northeast (NE) region: Pennsylvania, New York, New Hampshire, and Connecticut; Southeast (SE) region: Virginia, Maryland, Florida, North Carolina, and Alabama; Midwest (MW) region: Missouri, Illinois, and Michigan; Southwest (SW) region: Oklahoma, Kansas, and Texas; West (W) region: California.

Based on zip code information there was a wide geographic range of the participants ranging from New York to California across the Mid-Atlantic region, South, Upper Midwest, and Plains states. There were no participants from the northwestern part of the U.S. The majority (70%) were practicing in hospital-based practices, with the remaining participants roughly evenly distributed over private practices, community health centers, FQHC's, and other non-specified practice types.

The overwhelming majority of the participants (99%) were able to correctly identify the definition of health literacy used by the Institute of Medicine. Most participants agreed or strongly agreed that they could correctly identify participants with low health literacy, and this opinion was especially prevalent among residents more so than faculty (76% vs. 53%; *p* < .001). A minority of participants indicated that they were familiar with the term universal health literacy precautions, and there was no significant difference between residents and faculty (19% vs. 26%; *p* = .13). Only a small fraction of participants had ever used a health literacy assessment tool, and there was not a significant difference between residents and faculty (6% vs. 13%, *p* = .06). Many residents and faculty stated that they had received no training in health literacy, and there was no difference between the two groups (37% vs. 38%; *p* = .67). Notably, among residents there were no significant differences in response between interns and upper-level residents.

### Free Response Question #1

What have been some unexpected challenges in addressing health literacy as a part of your practice? Four themes emerged from the coding and consensus process: (1) time, (2) language, (3) limited training or resources, and (4) identifying low health literacy.

Most comments from providers mentioned some aspect of time as an unexpected challenge in addressing health literacy. Responses in this category focused on not having enough time to adequately address low health literacy in parents. Providers highlighted limited appointment times and busy schedules as challenges. They commented on the added time required for the incorporation of health literacy strategies. Responses pertaining to language highlighted the barrier that non-English–speaking families face. Providers expressed concern that interpreters may not always accurately translate and commented on the added difficulty of knowing if patients understand what they were being told. Responses within the limited training/resources theme demonstrated the lack of resources and training that providers perceive around health literacy. Comments included not having enough resources but also not knowing what resources would be most helpful. Within the theme identifying low literacy, residents and faculty expressed trouble identifying low literacy parents. They also emphasized that some families try to hide their low literacy level. Representative quotes are found in **Table 2**.

**Table 2:**
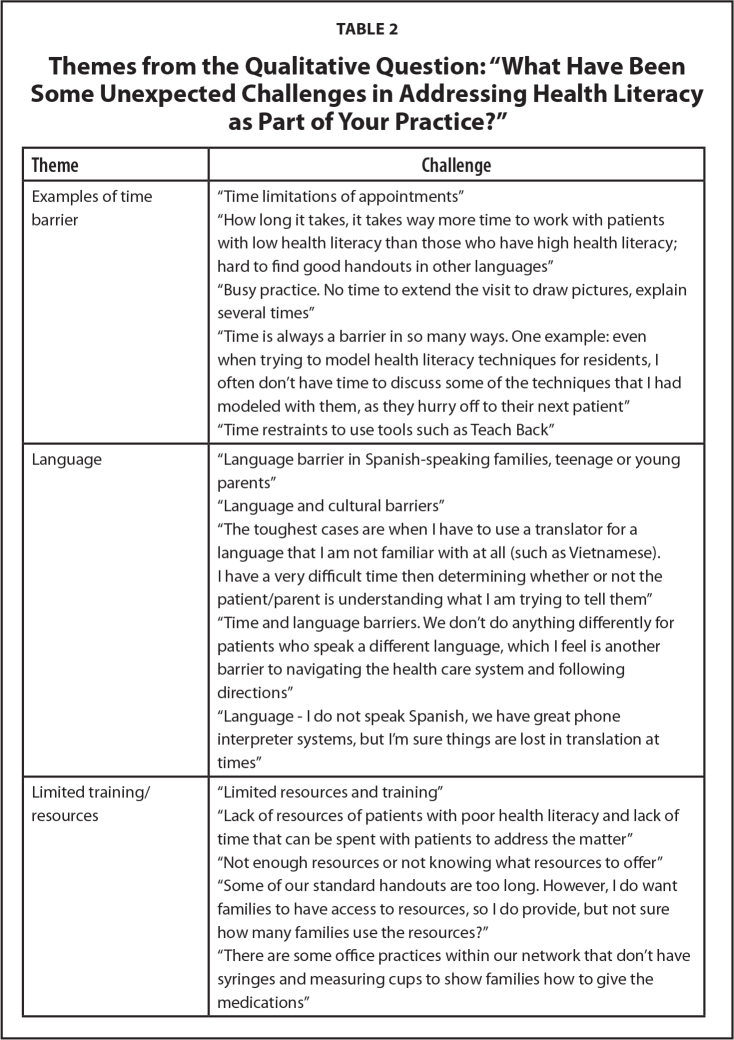

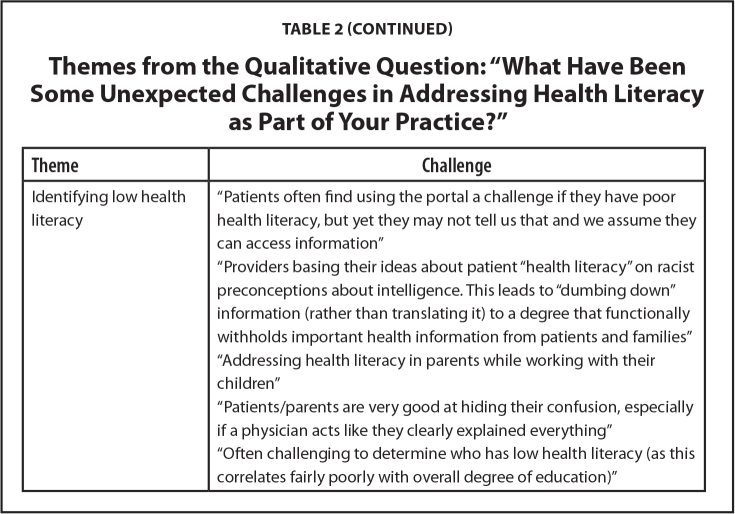
Themes from the Qualitative Question: “What Have Been Some Unexpected Challenges in Addressing Health Literacy as Part of Your Practice?”

**Theme**	**Challenge**

Examples of time barrier	“Time limitations of appointments”
	“How long it takes, it takes way more time to work with patients with low health literacy than those who have high health literacy; hard to find good handouts in other languages”
	“Busy practice. No time to extend the visit to draw pictures, explain several times”
	“Time is always a barrier in so many ways. One example: even when trying to model health literacy techniques for residents, I often don't have time to discuss some of the techniques that I had modeled with them, as they hurry off to their next patient”
	“Time restraints to use tools such as Teach Back”

Language	“Language barrier in Spanish-speaking families, teenage or young parents”
	“Language and cultural barriers”
	“The toughest cases are when I have to use a translator for a language that I am not familiar with at all (such as Vietnamese).
	I have a very difficult time then determining whether or not the patient/parent is understanding what I am trying to tell them” “Time and language barriers. We don't do anything differently for patients who speak a different language, which I feel is another barrier to navigating the health care system and following directions”
	“Language - I do not speak Spanish, we have great phone interpreter systems, but I'm sure things are lost in translation at times”

Limited training/resources	“Limited resources and training”
	“Lack of resources of patients with poor health literacy and lack of time that can be spent with patients to address the matter”
	“Not enough resources or not knowing what resources to offer”
	“Some of our standard handouts are too long. However, I do want families to have access to resources, so I do provide, but not sure how many families use the resources?”
	“There are some office practices within our network that don't have syringes and measuring cups to show families how to give the medications”

Identifying low health literacy	“Patients often find using the portal a challenge if they have poor health literacy, but yet they may not tell us that and we assume they can access information”
	“Providers basing their ideas about patient “health literacy” on racist preconceptions about intelligence. This leads to “dumbing down” information (rather than translating it) to a degree that functionally withholds important health information from patients and families”
	“Addressing health literacy in parents while working with their children”
	“Patients/parents are very good at hiding their confusion, especially if a physician acts like they clearly explained everything”
	“Often challenging to determine who has low health literacy (as this correlates fairly poorly with overall degree of education)”

### Free Response Question #2

Please elaborate on an experience with a patient/care-giver where health literacy was a barrier and you felt that it impacted care. Six themes emerged from the coding and consensus: (1) general literacy or obvious confusion, (2) indicating understanding when not present, (3) disease mismanagement, (4) resource use or nonuse, (5) language, and (6) fixed misconceptions.

Faculty and residents had a wide range of responses for how health literacy was a barrier influencing medical care. Most comments for the theme general literacy or obvious confusion emphasized families not understanding medical information and how this affected their children. Here, physicians noted that parents with illiteracy made providing care more challenging. The theme, indicating understanding when not present, highlighted how families can agree to things that they do not understand and how that has unintended consequences. The theme of disease mismanagement mostly centered around asthma care, missed appointments, and families not understanding their child's diagnosis and treatment plan. Physicians also commented on families not knowing how to use their child's medication correctly. For resource use and nonuse, comments included missing appointments from lack of transportation and not following up with referrals. The theme also highlighted the extra use of resources like the emergency department that families require when they do not understand their child's diagnosis, treatment, and medication regimens. Again, language emerged as a theme. Many faculty and residents remarked that language, especially in written form, caused significant barriers. For the theme, fixed misconceptions, comments spotlighted families not believing in the diagnosis or treatment plans such as routine vaccinations. Representative quotes are found in **Table 3**.

**Table 3:**
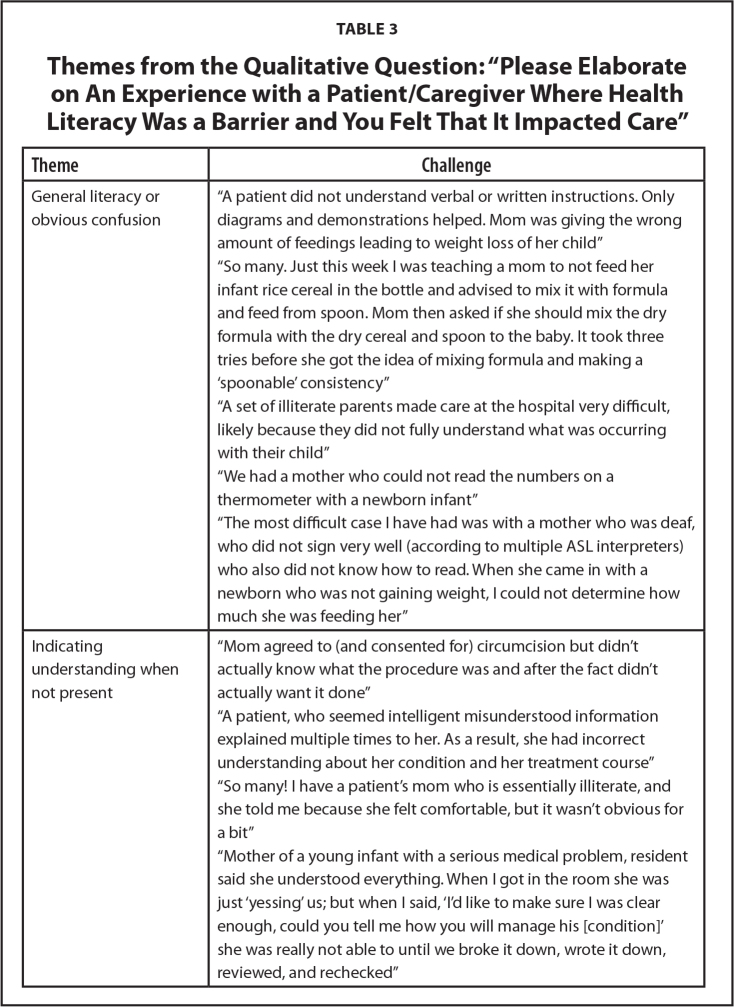

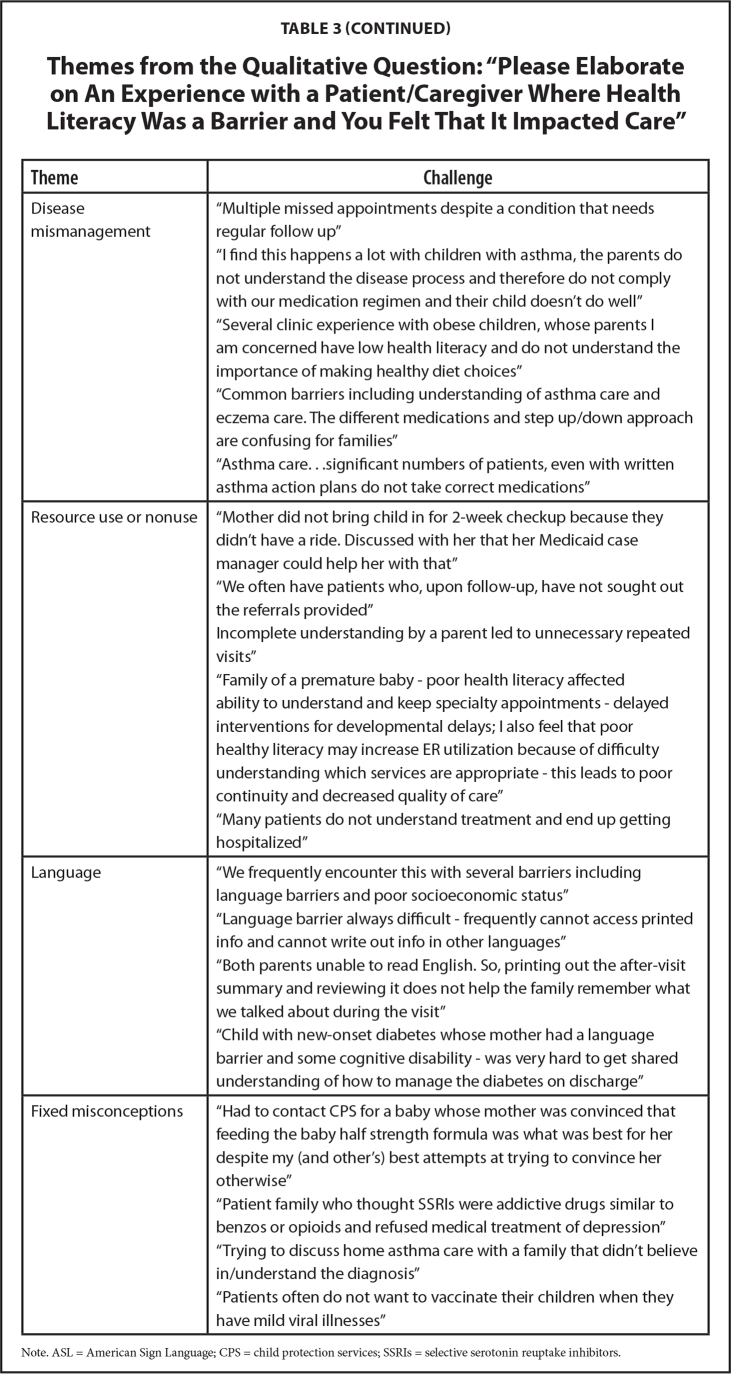
Themes from the Qualitative Question: “Please Elaborate on An Experience with a Patient/Caregiver Where Health Literacy Was a Barrier and You Felt That It Impacted Care”

**Theme**	**Challenge**

General literacy or obvious confusion	“A patient did not understand verbal or written instructions. Only diagrams and demonstrations helped. Mom was giving the wrong amount of feedings leading to weight loss of her child”
	“So many. Just this week I was teaching a mom to not feed her infant rice cereal in the bottle and advised to mix it with formula and feed from spoon. Mom then asked if she should mix the dry formula with the dry cereal and spoon to the baby. It took three tries before she got the idea of mixing formula and making a 'spoonable' consistency”
	“A set of illiterate parents made care at the hospital very difficult, likely because they did not fully understand what was occurring with their child”
	“We had a mother who could not read the numbers on a thermometer with a newborn infant”
	“The most difficult case I have had was with a mother who was deaf, who did not sign very well (according to multiple ASL interpreters) who also did not know how to read. When she came in with a newborn who was not gaining weight, I could not determine how much she was feeding her”

Indicating understanding when not present	“Mom agreed to (and consented for) circumcision but didn't actually know what the procedure was and after the fact didn't actually want it done”
	“A patient, who seemed intelligent misunderstood information explained multiple times to her. As a result, she had incorrect understanding about her condition and her treatment course” “So many! I have a patient's mom who is essentially illiterate, and she told me because she felt comfortable, but it wasn't obvious for a bit”
	“Mother of a young infant with a serious medical problem, resident said she understood everything. When I got in the room she was just ‘yessing’ us; but when I said, ‘I'd like to make sure I was clear enough, could you tell me how you will manage his [condition]’ she was really not able to until we broke it down, wrote it down, reviewed, and rechecked”

Disease mismanagement	“Multiple missed appointments despite a condition that needs regular follow up”
	“I find this happens a lot with children with asthma, the parents do not understand the disease process and therefore do not comply with our medication regimen and their child doesn't do well”
	“Several clinic experience with obese children, whose parents I am concerned have low health literacy and do not understand the importance of making healthy diet choices”
	“Common barriers including understanding of asthma care and eczema care. The different medications and step up/down approach are confusing for families”
	“Asthma care. . .significant numbers of patients, even with written asthma action plans do not take correct medications”

Resource use or nonuse	“Mother did not bring child in for 2-week checkup because they didn't have a ride. Discussed with her that her Medicaid case manager could help her with that”
	“We often have patients who, upon follow-up, have not sought out the referrals provided”
	Incomplete understanding by a parent led to unnecessary repeated visits”
	“Family of a premature baby - poor health literacy affected ability to understand and keep specialty appointments - delayed interventions for developmental delays; I also feel that poor healthy literacy may increase ER utilization because of difficulty understanding which services are appropriate - this leads to poor continuity and decreased quality of care”
	“Many patients do not understand treatment and end up getting hospitalized”

Language	“We frequently encounter this with several barriers including language barriers and poor socioeconomic status”
	“Language barrier always difficult - frequently cannot access printed info and cannot write out info in other languages”
	“Both parents unable to read English. So, printing out the after-visit summary and reviewing it does not help the family remember what we talked about during the visit”
	“Child with new-onset diabetes whose mother had a language barrier and some cognitive disability - was very hard to get shared understanding of how to manage the diabetes on discharge”

Fixed misconceptions	“Had to contact CPS for a baby whose mother was convinced that feeding the baby half strength formula was what was best for her despite my (and other's) best attempts at trying to convince her otherwise”
	“Patient family who thought SSRIs were addictive drugs similar to benzos or opioids and refused medical treatment of depression”
	“Trying to discuss home asthma care with a family that didn't believe in/understand the diagnosis”
	“Patients often do not want to vaccinate their children when they have mild viral illnesses”

Note. ASL = American Sign Language; CPS = child protection services; SSRIs = selective serotonin reuptake inhibitors.

## Discussion

The most striking findings in our study are the lack of knowledge regarding universal health literacy precautions, the widely held assumption that informal physician assessment of caregiver literacy is accurate, and the lack of reported education received regarding health literacy. These reported responses directly contradict available evidence and best practices. Although the term “universal health literacy precautions” may be specific to the AHRQ health literacy toolkit, the qualitative comments on barriers and unexpected challenges also highlights the lack of knowledge of basic health literacy strategies and techniques. Also, because most residents and faculty believe that they can effectively identify participants with low health literacy, the need for universal precautions seems irrelevant. These findings taken together suggest strongly there is a substantial number of patient visits in which appropriate communication is not taking place, and there are not adequate interventions in place to correct the situation.

There are ample data demonstrating that clinician assessment of health literacy of both patients and caregivers is poor (Bass et al., 2002; Cooper et al., 2018; Kelly & Haidet, 2007), yet most physicians and especially residents felt they could adequately assess health literacy. Universal health literacy precautions were designed to reduce reliance on physician assessment or health literacy assessment tools to stratify the types of information given to patients and to ensure that all patients and caregivers received adequate information. However, a disappointingly low number of residents and faculty had even heard of the concept. Coupled with the alarming result that approximately 2 of 5 residents and faculty had never received any training on health literacy; this indicates that there is enormous room for improvement in health literacy education. Focusing on one area such as Teach-Back techniques could be an effective approach for residency and faculty training programs. Teach-Back helps ensure that clinicians have adequately explained information clearly so that patients and their families understand the information that they have been given. Teach-Back has been shown to be an effective strategy for addressing health literacy and toolkits, and modules can easily be found for little or no cost online including the AHRQ Health Literacy Universal Precaution Toolkit (DeWalt et al., 2010). Concentrating on one strategy such as Teach-Back could allow residents and faculty to become proficient in that technique and become more time efficient, which would also address one of the barriers of time that many respondents identified as an obstacle.

Considering the above data, the frustration and confusion expressed by participants in their qualitative responses is unsurprising. Our qualitative results emphasize that providers in continuity clinics identify many varied challenges in addressing health literacy in clinical practice. Whereas the challenge of time is less easily addressed, standardized health literacy training could certainly be used to a greater extent. In addition, providers should be made aware of universal health literacy precautions, including the following strategies: plain language and clear communication, Teach-Back method, demonstrate/draw pictures, follow up with patients, brown bag medicine review, and addressing language differences. There were various identified challenges related to patients and families who do not speak English. Finding ways to promote improved language access and interpreter services can and should be a priority in pediatric continuity clinics. The effect of poor health literacy was most profoundly outlined in the responses from providers concerning examples of when they had experienced health literacy as a barrier in patient care. These real-life examples show the far-reaching consequences of unaddressed poor health literacy and emphasize the essential nature of addressing poor health literacy in a clinical setting.

## Study Strengths and Limitations

The response rate for our study was less than 50%, and although this may make our quantitative results difficult to generalize, it was noted during qualitative data analysis that saturation was reached in qualitative data collection. Thus, the qualitative results of this study appear to be a valid representation of our study population. In addition, membership of CORNET is program-based, so study recruitment requires a residency program representative to enroll in their program and disseminate the study to colleagues. It is possible that residency programs that place a greater emphasis on or interest in health literacy would be more inclined to participate. This could make the study findings more impressive, as it shows that even in potentially more interested programs, there are incorrect assumptions and practices and a general lack of knowledge on some subjects. Although we powered the study to test for differences between residents and faculty, we did not have sufficient power to confidently detect differences by level of training. However, given the overall low percentage of reported training, such differences would not add much to our conclusions.

The major strength of this study was its diversity of respondents. Participating programs were from a broad geographic distribution, and there was robust participation from all years of training and from faculty. The data serve as a critical needs assessment to drive educational interventions to improve resident and faculty knowledge of health literacy principles and strategies to improve communication with patients in the primary care pediatric setting. In follow up to this work, we plan to recruit residency programs to participate in the development and testing of a strategy to disseminate tools such as the AHRQ Health Literacy Toolkit to educate residents and faculty nationally. In addition to dissemination of the AHRQ Toolkit, we plan to use a quality improvement framework within the context of a learning collaborative.

## Conclusion

Physician knowledge of health literacy and the incorporation of associated best practices are essential to proper care of pediatric patients. Although almost every survey participant could define health literacy, the majority had never heard of universal health literacy precautions. Additionally, most participants made the dangerous assumption that they could accurately assess a caregiver's health literacy level, which prior research has shown to be false. These findings, paired with the evidence that health literacy training is not a standard part of pediatric residency programs, indicate that many providers may have risk factors for providing suboptimal care. Addressing these important issues in pediatric graduate medical education will foster more competent physicians and ultimately improve patient care.
